# Hypertension: adherence to treatment in rural Bangladesh – findings from a population-based study

**DOI:** 10.3402/gha.v7.25028

**Published:** 2014-10-20

**Authors:** Masuma Akter Khanam, Wietze Lindeboom, Tracey Lynn Perez Koehlmoos, Dewan Shamsul Alam, Louis Niessen, Abul Hasnat Milton

**Affiliations:** 1 Centre for Control of Chronic Diseases in Bangladesh, icddr,b, Mohakali, Bangladesh; 2 Centre for Clinical Epidemiology and Biostatistics, School of Medicine and Public Health, Faculty of Health and Medicine, The University of Newcastle, Callaghan, NSW, Australia; 3 Health Economics, Liverpool School of Tropical Medicine, Liverpool, UK; 4 International Health, Johns Hopkins School of Public Health, Baltimore, MD, USA

**Keywords:** adherence to treatment, hypertension, Bangladesh, village doctors, low-income country

## Abstract

**Background:**

Poor adherence has been identified as the main cause of failure to control hypertension. Poor adherence to antihypertensive treatment is a significant cardiovascular risk factor, which often remains unrecognized. There are no previous studies that examined adherence with antihypertensive medication or the characteristics of the non-adherent patients in Bangladesh.

**Objective:**

This paper aims to describe hypertension and factors affecting adherence to treatment among hypertensive persons in rural Bangladesh.

**Design:**

The study population included 29,960 men and women aged 25 years and older from three rural demographic surveillance sites of the International Center for Diarrheal Disease Research, Bangladesh (icddr,b): Matlab, Abhoynagar, and Mirsarai. Data was collected by a cross-sectional design on diagnostic provider, initial, and current treatment. Discontinuation of medication at the time of interview was defined as non-adherence to treatment.

**Results:**

The prevalence of hypertension was 13.67%. Qualified providers diagnosed only 53.5% of the hypertension (MBBS doctors 46.1 and specialized doctors 7.4%). Among the unqualified providers, village doctors diagnosed 40.7%, and others (nurse, health worker, paramedic, homeopath, spiritual healer, and pharmacy man) each diagnosed less than 5%. Of those who started treatment upon being diagnosed with hypertension, 26% discontinued the use of medication. Age, sex, education, wealth, and type of provider were independently associated with non-adherence to medication. More men discontinued the treatment than women (odds ratio [OR] 1.74, confidence interval [CI] 1.48–2.04). Non-adherence was greater when hypertension was diagnosed by unqualified providers (OR 1.52, CI 1.31–1.77). Hypertensive patients of older age, least poor quintile, and higher education were less likely to be non-adherent. Patients with cardiovascular comorbidity were also less likely to be non-adherent to antihypertensive medication (OR 0.79, CI 0.64–0.97).

**Conclusions:**

Although village doctors diagnose 40% of hypertension, their treatments are associated with a higher rate of non-adherence to medication. The hypertension care practices of the village doctors should be explored by additional research. More emphasis should be placed on men, young people, and people with low education. Health programs focused on education regarding the importance of taking continuous antihypertensive medication is now of utmost importance.

Worldwide, 13.5% of all premature deaths are attributable to high blood pressure (HBP) (1). Elevated blood pressure accounts for two-thirds and one-half of all cases of stroke and ischemic heart disease, respectively. Eighty percent of this burden occurred in low- and middle-income countries (2). Treatment reduces the risk of stroke by 30–41% and of coronary heart diseases by 22% (3). Despite the availability of effective treatments, studies have shown that in many countries, less than 25% of patients treated for hypertension achieve optimum blood pressure (4).

Detection, treatment, and control of hypertension are considered to be inadequate in many high-income countries; in low-income countries, where awareness about hypertension is generally low, the situation is even worse (5–8). A recent study in the Netherlands showed only a third of the individuals with hypertension were aware of their condition and among those aware about 60% were treated but only 42% had their blood pressure well controlled (9). Another study in China reported only 31% of the people with hypertension were treated and only 20% were controlled (10). In the United States, 65% of hypertensive individuals received treatment and nearly 50% were controlled (11). In Venezuela, only 4.5% of the treated patients had good blood pressure control (12). Poor adherence has been identified as the main cause of failure to control hypertension (4, 13). Poor adherence to antihypertensive treatment is a significant cardiovascular risk factor, which many a times remains unrecognized (4). Poor adherence to antihypertensive therapy increases the risk of stroke among the hypertensive individuals (14). The most important aims for the adequate control of hypertension are daily compliance and long-term adherence to therapy (15). Studies found that patients with hypertension tend to take less than half of their prescribed medications (16).

Previous research has shown that among elderly hypertensive patients taking medication, only 10% were well controlled (6). In a recent study among the elderly people in the Matlab Health and Demographic Surveillance Area, prevalence of hypertension is 50% among the elderly people of rural Bangladesh (17), but only 26% had control of their blood pressure (data not published). Information regarding current management practices for hypertension is scarce in rural Bangladesh. There is no existing study to examine adherence with antihypertensive medication and none has examined the characteristics of the non-adherent patients in Bangladesh. The aim of the study was to describe hypertension and the determinants of non-adherence to treatment among adult hypertensive persons in rural Bangladesh.

## Methods

### Ethical approval

Ethical approval for surveillance site activities has been obtained from the International Center for Diarrheal Disease Research, Bangladesh (icddr,b) review board and Human Research Ethics Committee, The University of Newcastle, Australia.

### Study sites

The study population was obtained from three rural demographic surveillance sites, Matlab, Abhoynagar, and Mirsarai. Table 1 describes the comparative characteristics of these three sites.

The three rural sites are similar in terms of population composition, density, household size, primary occupation, religion, and their disease profile; and also in area characteristics (18–20). Rural Bangladesh is mostly plain land and riverine area. In general, the public health care delivery is very homogenous across the country. The services of community health workers, village doctors, and so on are very similar across the sites. Furthermore, there is no specific initiative from any public or private organizations to date, regarding managing chronic conditions *per se* hypertension, in these sites. The population under research was from icddr,b surveillance sites where icddr,b has been maintaining contact with the population in a very similar fashion across the sites for many years: in Matlab since the early 1960s, and in Mirsarai and Abhoynagar since the early 1980s. The sampling design of the Health and Sociodemographic Surveillance System (HDSS) was a stratified two-stage sampling; unions were stratified initially. Unions are administrative subunits in Bangladesh with a population of approximately 20,000 to 30,000. Each unions were randomly selected. Households served as the second stage sampling units. A systematic random sampling technique was applied to select the sample households.

Among sample households, all household members were identified and listed to collect basic socioeconomic and demographic data, and a unique identification number was assigned to each individual. At regular 90-days intervals, one female interviewer visited each household to collect data on demographic and other programmatic events. HDSS is a cohort study, but this study presents a cross-sectional analysis of base-line data regarding hypertension medication adherence in HDSS population.

### Study population

The study population was limited to individuals aged 25 years and above. Data was collected in a cross-sectional survey during the year 2009, for a 2-month period at each site. The study population was sampled by door to door survey, during the regular surveillance rounds. In Abhoynagar and Mirsarai, an attempt was made to include respondents from all households, and in Matlab about 6,400 individuals were interviewed, evenly distributed across the research area.

Because information was collected during regular household visits, only those present during the visit, and meeting the age criterion were included. This resulted in a biased selection of respondents, with an overrepresentation of women. To adjust for this bias, the research population was weighted to match the relative age–sex distribution of the populations each of the surveillance sites. In order to give equal weight to the three rural surveillance sites, the sampled population for Matlab was inflated to meet the sample size of the other two sites.

Data were collected during the regular visits and only those present at the time of interview were included. We did not track the non-response rate as the sample was not drawn from our defined HDSS surveillance population. However, a typical estimate of absenteeism of a given household is approximately less than 5% in the HDSS surveillance sites (21). In total 29,960 individuals were included.

### Data collection

Trained research assistants conducted the face-to-face interviews in Bangla using a two-part questionnaire on chronic disease lifestyle risk factors and management. The questionnaire was translated to English and then back translated to Bangla to check the consistency of the meaning. We then piloted the questionnaire with 10 people from an area similar to but other than the study sites, to see the understandability of the questionnaire, language, and so on. This pretested structured questionnaire collected information on 11 prespecified chronic conditions regarding diagnoses, initial treatment, current treatment, and health care provider. Respondents were asked ‘Have you ever been told by any of the following personnel: MBBS doctor, specialized doctor, nurse, health worker, paramedic (Medical assistant/sub assistant community medical office), village doctor/quack, homeopath, kabiraj, or pharmacy man that you have any of the following medical conditions: hypertension, diabetes, abnormal blood lipids, overweight, chronic bronchitis, heart attack, angina/coronary heart disease, stroke, asthma, oral cancer, lung cancer and others’. Respondents then needed to identify the most recent provider of the diagnoses. Diagnosis was solely based on self-reporting; details such as symptoms, signs, or lab tests were not included. All the information collected in this study was self-reported. We only reported about the hypertension in this paper.

### Dependent and independent variables

The dependent variable for this study was non-adherence to antihypertensive treatment, which we have defined as discontinuation of medication at the time of interview, when treatment was received at initial diagnosis. It is categorized in to ‘yes’ and ‘no’. The independent variables were age, sex, education, asset index, comorbidity, and health care provider. The individual sociodemographic factors were derived from preexisting surveillance data.

### Definitions

#### Hypertension

Hypertension was diagnosed in this study by asking the respondents: ‘Have you ever been told by any of the following personnel: MBBS doctor, specialized doctor, nurse, health worker, paramedic (Medical assistant/sub assistant community medical office), village doctor/quack, homeopath, kabiraj, or pharmacy man that you have hypertension?’ Hypertension diagnosis is not based on blood pressure measurements; rather we used face-to-face interviews with a structured questionnaire. For the validity of the diagnosis we have asked who provided the diagnosis and to strengthen the diagnostic work we have also asked if they were taking any medication for their hypertension diagnosis. Community health workers or informal health providers or unqualified providers, whatever we want to call, they provide extensive health services in low-income countries. Unqualified providers are crucial to Bangladesh's pluralistic health care system. Ninety-two percent of the unqualified providers (Bangladesh is divided in 64 districts, second tier for administrative purposes) have district level training and more than 96% have specific training on hypertension (22).

#### Asset index

The asset index was assessed based on household assets and housing characteristics, including bed, mattress, quilt, cooking pots, watch, chair, clothing cabinet, radio, television, bicycle, boat, cows, and electricity. Using a variable reduction technique, these assets and characteristics were combined into a single variable. Details about the calculation of the asset index can be found in other publications from the Matlab HDSS (23). After ranking this variable from low to high, households were divided into five equally sized groups, the poverty quintiles. This procedure was repeated for each site; household stratification did not account for possible poverty/wealth differences between sites.

#### Comorbidity

Comorbidity was defined when respondents reported being diagnosed with cardiovascular diseases, other than hypertension, abnormal blood lipids, or overweight. Diagnosing provider was categorized as qualified doctors (MBBS and specialized physicians) and unqualified providers (nurses, health workers, paramedics, village doctors, homeopath, kabiraz/spiritual healers, and pharmacy).

### Statistical analyses

Data was presented with mean (standard deviation, SD) for continuous variables and with proportion for categorical variables. The overall and sex-specific prevalences of hypertension were calculated. The study participants were divided into four age groups (<40, 40–49, 50–59, and 60+ years). Categorical variables were compared by chi-square statistics.

Univariate regression analysis was performed to identify the factors that were associated with non-adherence. Any factor that provided a univariate *p*-value <0.05 was entered into a multiple regression model. Logistic regression analyses were performed to estimate odds ratios (OR) and 95% confidence intervals (CI) of non-adherence associated with various factors, with and without adjustment for other explanatory variables. SAS (Version 8) Statistical software was used for the analysis.

## Results

The study population comprised 52.6% women, mean age was 44.6 years, with 17% belonging to the age group of 60 years and above. Those who had no formal education amounted to 43.7%. The prevalence of hypertension was 13.67%, higher for women (14.8%) than men (8.9%) (*p*<0.0001). Prevalence of hypertension increased with age (5.9% in the youngest age group compared to 23.1% in the oldest age group; *p*<0.0001), higher among the least poor quintile compared to the poorest quintile (20.6% vs. 6.3%, *p*<0.0001), and higher with increasing education (no formal education 12.2% vs. highest education 16.9%) (*p*<0.0001) (Table 2).

**Table 1:** Characteristics of the three study sites

Characteristics	Matlab	Mirsarai	Abhoynagar
Rural/urban	Rural	Rural	Rural
Location	55 km southeast of capital city (nearly central in the country)	Southeastern part of the country	Southwestern part of the country
Average household income	Per capita household income in 2001 approx. 9,000 BDT	8,040 BDT/month Median 6,000 BDT	5,609 BDT/month Median 4,000 BDT
Total surveillance population in 2009	113,186	39,025	34,717
Sample population			
Men	1,648	3,584	4,435
Women	4,725	8,410	7,158
Disease profile is predominantly	Fever, digestive disturbance, and respiratory disease	Fever, digestive disturbance, and respiratory disease	Fever, digestive disturbance, and respiratory disease
Population Density	1,100 persons/km^2^	1,067 persons/km^2^	1,067 persons/km^2^
Household size	5.1 persons	5.4 persons	5.4 persons
Primary occupation	Cultivation	Cultivation	Cultivation
Religion	Muslims (90%)	Muslims (>80%)	Muslims (>80%)

Only 58% of men and 51.1% of women with hypertension were diagnosed by qualified doctors. Among the unqualified providers, village doctors diagnosed 37% of the men and 42.7% of the women (Table 3).

**Table 2:** Characteristics of the study population (*n*=29,960) by hypertension status

		Hypertension		
		
Characteristics	No. of participants	Present	Absent	*p*
(*n*)	29,960	4,097	25,863	
Age groups, years (%)				
< 40	43.1	5.9	94.1	
40–49	23.9	11.8	88.2	<0.001
50–59	16.0	17.3	82.7	
60 and above	17.0	23.1	76.9	
Mean age (std)	44.6 (15.8)	52.8 (14.3)	43.6 (14.1)	<0.001
Sex (%)				
Male	47.4	8.9	91.1	<0.001
Female	52.6	14.8	85.2	
Level of education (%)				
No education	43.7	12.1	87.9	
Primary (1–5 years)	26.9	12.0	88.0	
Secondary (6–10 years)	24.5	11.9	88.1	0.727
Higher (11+ years)	4.8	12.8	87.2	
Years of education (std)	3.6 (4.4)	3.7 (4.0)	3.6 (4.0)	0.201
Asset Index (%)				
Poorest 20%	16.3	6.5	93.5	
Poorer 20%	18.2	9.0	91.0	
Middle 20%	20.0	10.5	89.5	
Less poor 20%	22.0	13.4	76.6	<0.001
Least poor 20%	23.6	18.9	81.1	

**Fig. 1:**
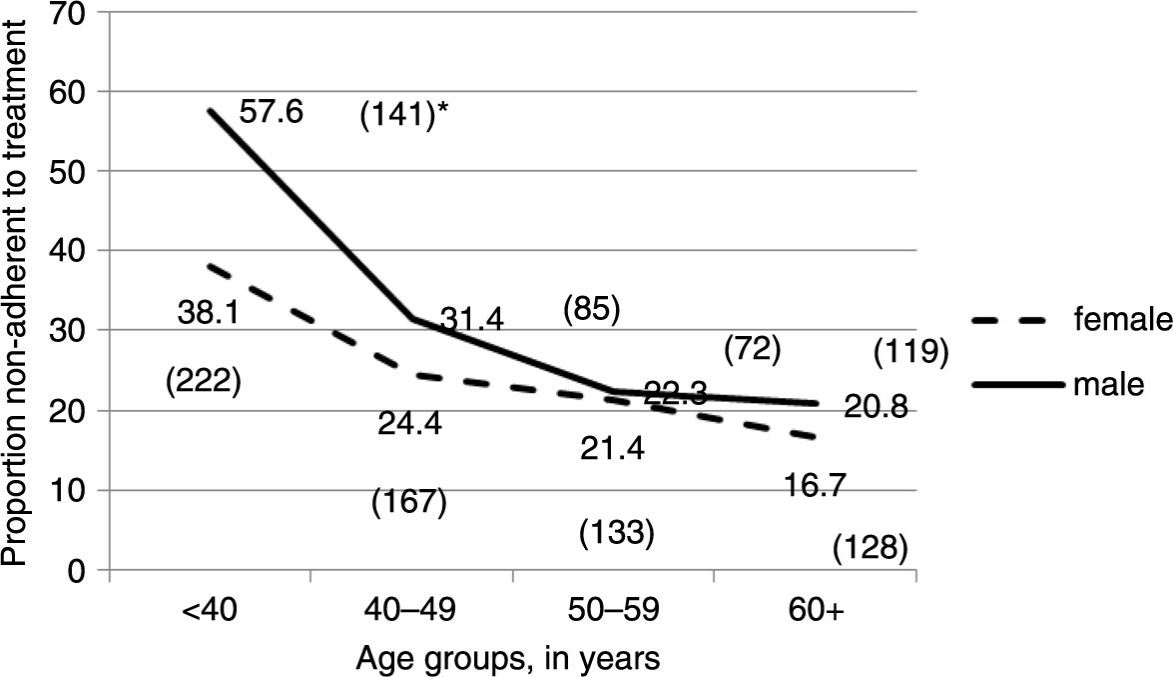
Percentage of people non-adherent to treatment by age group and sex, in rural Bangladesh, 2009. *Absolute numbers of samples are shown in the parenthesis.

**Fig. 2:**
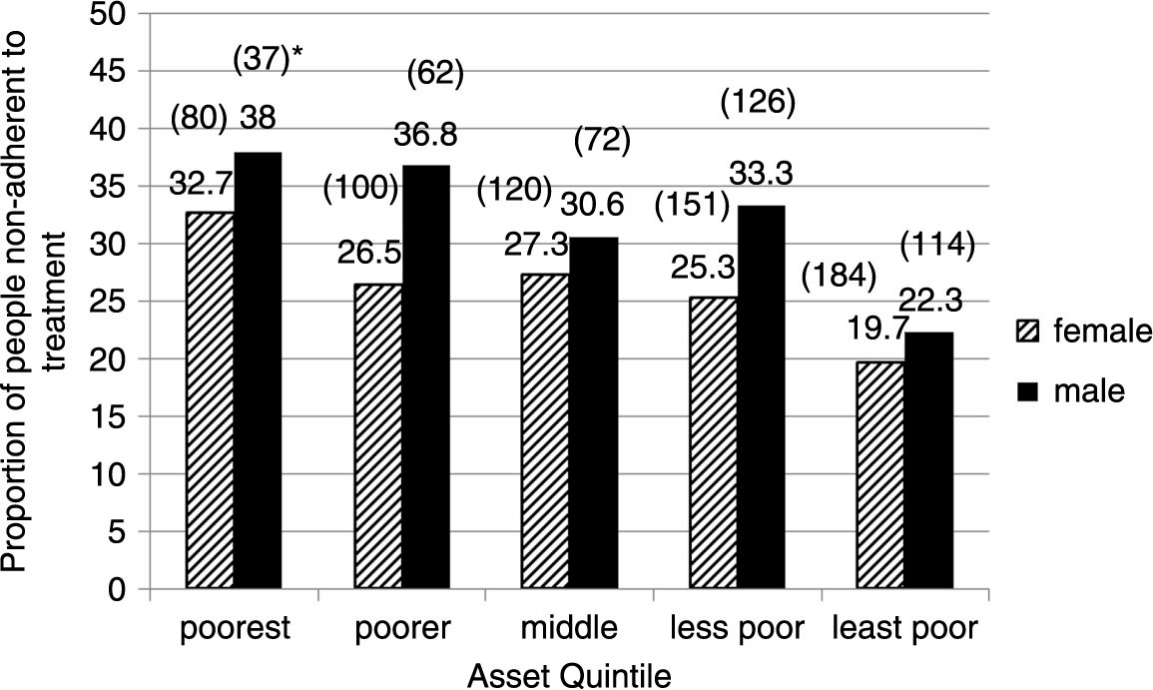
Percentage of people non-adherent to treatment by sex and asset quintile. *Absolute numbers of samples are shown in the parenthesis.

The proportion of people non-adherent to treatment was 26.2% in the study population. Non-adherence to treatment was higher among men (29.2%) than women (24.3%) (*p*<0.0001). Non-adherence to treatment decreased with age (*p*<0.0001) (Fig. 1). Non-adherence was less common among the wealthy people, both for men and women (Fig. 2).

Age, sex, education, wealth, comorbidity, and type of providers were independently associated with non-adherence to antihypertensive medication. More men were non-adherent to the treatment than women (OR 1.67, CI 1.42–1.97). Non-adherence to medication was greater when hypertension was diagnosed by unqualified providers (OR 1.46, CI 1.31–1.77). People of older age, higher education, and with most wealth were less likely to be non-adherent. Those who reported cardiovascular comorbidity (*angina, heart attack, or stroke*) were more likely to be compliant to medication (OR 0.78, CI 0.64–0.97) (Table 4).

**Table 3:** Hypertension diagnosed by type of health care providers and sex in rural Bangladesh

	Diagnosis of hypertension (%)			
	
	Male	Female	Total	*p*
MBBS doctor	48.8	44.5	46.1	0.007
Specialized doctor	9.1	6.5	7.4	0.002
Nurse	0.0	0.1	0.1	0.168
Health worker	0.3	0.3	0.3	0.858
Paramedic	3.3	4.5	4.1	0.054
Quack/village doctor	37.0	42.7	40.7	0.001
Homeopath	0.6	0.6	0.6	0.931
Kabiraz/spiritual healer	0.1	0.0	0.0	0.418
Pharmacy	0.7	0.7	0.7	0.869

**Table 4:** Unadjusted and adjusted odds ratio (OR) and 95% confidence intervals (CI) of discontinuation of (non-adherence to) hypertension treatment in rural Bangladesh

	Non-adherence to hypertension treatment	
	
Characteristics (*n*)	Unadjusted OR (95% CI)	Adjusted OR (95% CI)
Age groups, years		
< 40 (363)	1	1
40–49 (252)	0.46 (0.38–0.56)	0.45 (0.36–0.55)
50–59 (205)	0.36 (0.29–0.44)	0.34 (0.27–0.42)
60 and above (248)	0.29 (0.24–0.35)	0.27 (0.22–0.33)
Sex		
Female (651)	1	1
Male (417)	1.29 (1.12–1.49)	1.67 (1.42–1.97)
Education		
No education (471)	1	1
Primary (1–5 years) (305)	1.08 (0.91–1.27)	1.00 (0.83–1.20)
Secondary (6–10 years) (255)	1.11 (0.84–1.20)	0.80 (0.65–0.99)
Higher secondary (11+ years) (31)	0.52 (0.35–0.78)	0.38 (0.25–0.60)
Asset Index		
Poorest 20% (117)	1	1
Poorer 20% (162)	0.83 (0.62–1.10)	0.84 (0.63–1.14)
Middle 20% (192)	0.75 (0.57–0.99)	0.87 (0.65–1.16)
Less poor 20% (277)	0.77 (0.59–1.00)	0.89 (0.68–1.18)
Least poor 20% (297)	0.50 (0.38–0.64)	0.69 (0.52–0.92)
Comorbidity		
No (919)	1	1
Yes (149)	0.62 (0.51–0.76)	0.78 (0.64–0.97)
Diagnosing provider		
Qualified doctors (461)	1	1
Unqualified providers (605)	1.78 (1.55–2.05)	1.46 (1.31–1.77)

## Discussion

This is the first study reporting prevalence and correlates of non-adherence to antihypertensive treatment in rural Bangladesh. The prevalence of hypertension is 13.67% (95% CI 13.29–14.07) in this study. A recent study conducted in the same areas of Bangladesh reported that the prevalence of hypertension varied from 9.3 to 24.1% (24). A recent systematic review and meta-analysis of studies between 1995 and 2010 showed the pooled prevalence of hypertension in Bangladesh was 13.7% (overall prevalence estimate ranged between 9 and 22.2%) (25). Although we have collected self-reported information, the prevalence of hypertension reflects the magnitude of hypertension in rural Bangladesh very well. We have found that the prevalence of hypertension is significantly higher for women compared to men, consistent with other studies in rural Bangladesh. (26). Our finding that the prevalence is rising with increasing wealth is consistent with findings from other rural areas in Bangladesh (27).

We have found that more than 26% of the study population is non-adherent to antihypertensive treatment. In developed countries, adherence among patients suffering from non-communicable diseases averages only 50% (28). In China and the Gambia, only 43 and 27% of patients with hypertension adhere to their antihypertensive medication regimen, respectively (29, 30). In developing countries, the magnitude of poor adherence is assumed to be higher given the scarcity of health resources and difficulties in access to health care (31).

The reasons for poor adherence have been studied extensively in the West. Two of the most important factors contributing to poor adherence are the asymptomatic and lifelong nature of the disease. Other potential determinants of adherence may be related to demographic factors such as age and education, the patient's understanding and perception of hypertension, the health care provider's mode of delivering treatment, and the relationship between patients and health care professionals (32, 33). Data from the developing world, in this regard are scanty, with details of the prevalence of the condition has just beginning to emerge.

We have found that men in rural Bangladesh are most likely to discontinue the treatment. Similar findings were observed in other studies (34); however, opposing findings have also been reported (35). Our study showed that young hypertensive patients are less likely to continue antihypertensive treatment, in line with other findings (34–36). As the symptoms go unnoticed, young individuals may not pay much attention to the importance of continuing medication. This needs more exploration as an area of intervention. Sex and age are affecting adherence to treatment as in other diseases too (37).

Poor socioeconomic status and low education are important factors for poor adherence (38–40); our findings are in line with these evidences. Although the exact cause could not be revealed in this study, people with higher education and more wealth may be more health conscious and knowledgeable about the impact of poor control of HBP. Studies found that non-adherent patients with lower income and less education suffered more from stroke (14). Financial constraints could be a factor in continuing lifelong medication for hypertension treatment (32).

We have found that in the presence of cardiovascular comorbidities (vascular diseases, abnormal lipids, and overweight) the odds of non-adherence to antihypertensive medication are reduced. The reason could be numerous; patients with comorbidities visit health care providers more frequently, they pay more attention to their health conditions, and also are more likely to go to a qualified health care provider and therefore more likely to continue medication in general. Specific reasons for this population are yet to be revealed as aspects of multimorbidity and polypharmacy are underresearched in Bangladesh.

In this study, non-adherence is more when hypertension was diagnosed by village doctors. The influence of factors related to the health care provider on adherence to therapy for hypertension has not been systematically studied. One important factor is probably lack of knowledge and training for health care providers on managing hypertension *per se* chronic diseases (41, 42). These factors may contribute to the fact of more than 40% of people being diagnosed as hypertensive by the village doctors.

We have mainly focused on demographic and economic factors such as age, sex, education, and wealth, some of which are results of social determinants, that influence adherence to treatment. This study has highlighted the importance of health care providers in patient adherence to antihypertensive therapy, and the extent of poor adherence, especially when unqualified providers, for example, village doctors, play a prominent role in diagnosing the condition. We have used the education and asset index data, as in many earlier reports, to shed some light on the financial aspects of this study outcome, as there is a positive correlation between educational level and income. Unfortunately, we do not have any data about the cost borne by the patients of this study; neither do we have any data regarding usual cost of antihypertensive drugs. However, a publication on availability and affordability of essential medicines for chronic diseases in low-income countries mentioned that median price ratio is between 1.14 and 1.31 in Bangladesh (this ratio compares a medicine's median price to its international reference price) (43).

Several limitations should be mentioned. We have used the self-reported information because this is easy, economical, and has long been used as an epidemiological tool. This may be subject to recall bias and possibility of underestimation of actual prevalence. We have a robust phenomenon in place, our surveillance system. The surveillance people are in acquaintance with routine questionnaires and interviewers, decreasing the likelihood of reporting bias. In checking for the validity of hypertension diagnosis, participants were not only asked about the providers of the diagnosis but also asked about taking antihypertensive medication. However the focus of this paper was mainly the adherence to treatment for the hypertensive patients. There are more validated measures of medication adherence, where objective measures are used to specify the adherence. This is the first of its kind study on adherence to antihypertensive treatments in Bangladesh, and conducted on surveillance population.

Among the several strengths, this study covers a wide geographical area, provides initial steps of nationally representative data, and includes large sample size that supports the accuracy of our findings. HDSS collect information from whole communities over extended time periods, which more accurately reflect health and population problems in low- and middle-income countries. HDSS provides a reliable sampling frame and a high response rate is usually observed.

## Conclusions

Although village doctors make 40% of hypertension diagnoses, their treatments are associated with a higher rate of discontinuation. The hypertension management practices of the village doctors should be explored in subsequent research. More research is needed for better understanding of the determinants of adherence, also to find out the reasons for non-adherence to treatment particularly among men and young people. Accordingly, effective interventions need to be developed to properly manage hypertension and chronic diseases in general.
